# Editorial: (Osteo)sarcopenia & sarcopenic obesity, volume II

**DOI:** 10.3389/fendo.2023.1309645

**Published:** 2023-11-06

**Authors:** Ifigenia Kostoglou-Athanassiou, Lambros Athanassiou, Panagiotis Athanassiou, Stefano Masiero, Yannis Dionyssiotis

**Affiliations:** ^1^ Department of Endocrinology, Asclepeion Hospital, Voula, Athens, Greece; ^2^ Department of Rheumatology, Asclepeion Hospital, Voula, Athens, Greece; ^3^ Department of Rheumatology, St. Paul’s Hospital, Thessaloniki, Greece; ^4^ Rehabilitation Unit, Department of Neuroscience, University of Padova, Padua, Italy; ^5^ Physical Medicine and Rehabilitation School, University of Padova, Padua, Italy; ^6^ Spinal Cord Injury Rehabilitation Clinic, General University Hospital Patras, Rio Patras, Greece

**Keywords:** sarcopenia, sarcopenic obesity, exercise, diagnosis, osteoporosis

Sarcopenia is characterized by the presence of low muscle mass, loss of muscle strength and compromised function and is recognized today as a characteristic of progressing age as well as an accompanying characteristic of many disease states (1–3). Sarcopenia is recognized today as a major determinant of poor health related to various comorbidities and its relationship with mortality is actively investigated (4–6). Our Research Topic aimed to identify various comorbidities related to sarcopenia and to identify the effect of exercise on sarcopenia. Sarcopenia may accompany obesity in the concept of sarcopenic obesity, a state characterized by many comorbidities and compromised quality of life (7, 8). Liu et al. investigated the role of obesity in sarcopenia and the optimal body composition to prevent against sarcopenia and obesity. They found a positive relationship between skeletal muscle mass and absolute fat mass but negative association with appendicular fat mass. Obesity was found to be a risk factor for sarcopenia.

Sarcopenia may accompany osteoporosis, may lead to falls and this combination may increase mortality (9, 10). In a study performed in China involving 9,006 individuals with a follow-up of over 7 years it was shown that sarcopenia is accompanied by an increased mortality risk Xiong et al. In particular, the odds ratio of sarcopenia for 7-year mortality was 1.41, whereas for severe sarcopenia the odds ratio was even greater. The study underlined the significant association of sarcopenia with mortality and stressed that low-hand grip and usual walking speed are significant indicators of mortality risk at least within the Chinese population. In a study performed in Italy Maccarone et al. investigated the prevalence of sarcopenia in a cohort of elderly patients, aged over 65, who were cared for in a rehabilitation center and had musculoskeletal complains. They found a high percentage of patients with overt sarcopenia and approximately 10% with severe sarcopenia. Patients with severe sarcopenia had lower body mass index (BMI) and in the assessment of nutritional status they rated low. Liu et al. examined the effect of sarcopenia, osteoporosis and osteosarcopenia on spine fractures in American adults with prediabetes by using data from the NHANES study 2009 to 2018. People with prediabetes were more likely to develop sarcopenia than normal glucose tolerance subjects, while there was no significant increase of osteoporosis in prediabetes. Skeletal muscle mass was independently associated with osteoporosis in prediabetes adults. Sarcopenia and osteoporosis were positively associated with spine fracture in prediabetes.

In a study performed in West China, Xiang et al. explored the prevalence of sarcopenia, its association with osteoporosis and its effect on survival in patients on hemodialysis. In a group of 209 adult patients undergoing hemodialysis sarcopenia was diagnosed in 37.3%. Age, female sex, diabetes, serum magnesium and BMI were found to be independently associated with sarcopenia. The prevalence of osteosarcopenia was 22.3% and it was independently associated with all-cause mortality. They found that patients undergoing hemodialysis had a high incidence of sarcopenia and osteosarcopenia, the latter having a powerful association with mortality. Gong et al. investigated the relationship between lean body mass and cognitive function in old adults. They used data from the National Health and Nutrition Examination Survey (NHANES) 2011-2014. Their findings showed an association between predicted lean body mass and cognitive dysfunction in information processing speed.

Liu et al. investigated the relationship between COVID-19 and sarcopenia in a bidirectional Mendelian randomization analysis. Evidence suggested that COVID-19 patients were prone to skeletal muscle loss while sarcopenia may be associated with susceptibility, hospitalization, and severity of COVID-19. Using genetic data, the study explored the causal relationship between COVID-19 and sarcopenia related traits, but the results indicated that there was no such causal relationship. In an effort to identify the relationship between non-alcoholic fatty liver disease and sarcopenia Xu et al aimed to identify co-expressed genes in non-alcoholic fatty liver disease and sarcopenia. They conducted a complete transcription pattern mapping to identify core genes underlying biological mechanisms which regulate aging in non-alcoholic liver disease and sarcopenia patients.

Rosas-Carrasco et al. developed and validated a short new scale for the screening of sarcopenia, which they named Sarcopenia Geriatric Scale (SARCO-GS). The short scale was developed to be affordable, easy and accessible at all types of clinical settings and was found to be sensitive and to adequately predict functional dependence. The scale includes 7 items, five subjective questions and two measurements of strength and muscle mass. After validation, the scale, which was developed in Mexico, was adapted to English. Khalafi et al. performed a systematic review and meta-analysis of the effect of exercise training on body composition outcomes in postmenopausal women. They searched the main databases of medical literature, PubMed, Web of Science, CINAHL and Medline for randomized controlled trials on the effect of exercise training in postmenopausal women. The results showed that exercise training increased muscle mass and volume, muscle fat free mass and body and visceral fat and waist circumference. The results of the meta-analysis indicated that exercise training may improve body composition in postmenopausal women and that the combination of aerobic and resistance training may be an effective strategy for the improvement of body composition in the postmenopausal period.

Within our Research Topic we investigated further the relationship of sarcopenia with obesity, osteoporosis and spinal fractures and confirmed the positive relationship between sarcopenia, obesity, osteoporosis and spinal fractures. The relationship of sarcopenia with cognitive dysfunction was investigated and a positive correlation between sarcopenia and cognitive dysfunction was observed. A short new scale which aimed to be accessible at all clinical settings was developed for the screening of sarcopenia. The findings from the manuscripts included in the Research Topic underline the importance of various comorbidities associated with sarcopenia and the importance of exercise in its management. We feel the need to extend our gratitude to all the participants and contributors to the research projects and the papers included in this Research Topic and we are hopeful that the information presented will aid to the advancement of clinical practice and inspire further innovations in the future.

## Author contributions

IK-A: Investigation, Writing – original draft, Writing – review & editing. LA: Conceptualization, Writing – original draft, Writing – review & editing. PA: Investigation, Software, Writing – original draft. SM: Supervision, Validation, Writing – review & editing. YD: Methodology, Supervision, Validation, Writing – original draft, Writing – review & editing.
